# Associations between apolipoprotein CIII concentrations and microalbuminuria in type 2 diabetes

**DOI:** 10.3892/etm.2014.1830

**Published:** 2014-07-07

**Authors:** ZHI-JUAN HU, LU-PING REN, CHAO WANG, BING LIU, GUANG-YAO SONG

**Affiliations:** 1 Department of Nephrology, Hebei General Hospital, Shijiazhuang, Hebei 050051, P.R. China; 2 Department of Endocrinology, Hebei General Hospital, Shijiazhuang, Hebei 050051, P.R. China

**Keywords:** type 2 diabetes mellitus, diabetic nephropathy, apolipoprotein CIII, microalbuminuria

## Abstract

Microalbuminuria (MAU) is a strong predictor of diabetic nephropathy (DN), which is the main cause of morbidity and mortality in patients with diabetes mellitus (DM). Dyslipidemia exists in the majority of patients with DM and contributes to micro- and macrovascular complications associated with DM. Apolipoprotein CIII (apoCIII) is an inhibitor of the activity of lipoprotein lipase, which metabolizes triglyceride (TG) in very low-density lipoprotein (VLDL) and facilitates its clearance from plasma. The aim of the present study was to investigate the associations between apoCIII and MAU and the effects of atorvastatin in type 2 diabetes. In total, 120 subjects were divided into type 2 diabetes and type 2 DN groups, while 60 healthy subjects were selected as controls. The patients with DN were administered 20 mg atorvastatin daily for 16 weeks. Blood pressure, body mass index (BMI) and levels of HbA1c, FBG, TG, VLDL-cholesterol (VLDL-C), apoCIII and MAU were markedly elevated in the type 2 diabetes and type 2 DN groups compared with those in the control group (P<0.01), while high-density lipoprotein-cholesterol (HDL-C) levels were decreased significantly (P<0.01). All patients with type 2 DN showed significantly elevated blood pressure, apoCIII levels, MAU, course of the disease and rate of stroke and retinopathy compared with the patients with type 2 diabetes (P<0.01). MAU was significantly positively correlated with the course of the disease, systolic blood pressure, diastolic blood pressure, BMI and HbA1c, FBG, TG, total cholesterol, low-density lipoprotein-cholesterol, VLDL-C and apoCIII levels (P<0.05), whereas negatively correlated with HDL-C levels (*r*=−0.194, P=0.020). Logistic regression analysis showed that apoCIII levels were independently associated with MAU (odds ratio, 1.100; 95% confidence interval, 1.037–1.153; P<0.001). Atorvastatin improved the lipid profile and MAU in patients with type 2 DN (P<0.01). Therefore, the present study demonstrated that an independent positive correlation exists between the levels of apoCIII and MAU in patients with type 2 diabetes. Furthermore, atorvastatin may be used to improve the lipid profile and MAU in type 2 DN.

## Introduction

Diabetic nephropathy (DN) is a major cause of end-stage renal disease (ESRD). One of the early markers of DN and vascular disease in patients with diabetes is the presence of microalbuminuria (MAU). Without specific intervention, 20–40% of type 2 diabetic patients with MAU progress to overt nephropathy (1). Therefore, screening for MAU and timely therapeutic intervention has become the standard in care worldwide.

Plasma triglyceride (TG) levels are increased in patients with DN. The abnormal synthesis and clearance of TG and the lipoprotein particles associated with TG may be involved in the development of DN (2). There is evidence suggesting that TG-rich lipoprotein (TRL) particles predominantly containing apolipoproteins (apos) E, C and B may be major promoters of DN (2). In patients with DN, plasma TG levels may increase, partly due to the reduced activity of hepatic lipase (HL) and lipoprotein lipase (LPL), which hydrolyze TG (3).

ApoCIII is a protein composed of 79 amino acid residues that is predominantly synthesized in the liver, although synthesis also occurs in the intestine. ApoCIII is an exchangeable protein moiety between chylomicron remnants, very low-density lipoproteins (VLDLs) and high-density lipoproteins (HDLs) (4,5). In the blood circulation, ApoCIII is associated mainly with TRLs, HDLs and, to a lesser extent, low-density lipoproteins (LDLs) (6–8). Total plasma apoCIII levels have been found to be key determining factors of serum TG levels. Studies involving gene variation and circulating protein levels have implicated apoCIII as a risk factor for cardiovascular disease (9–12). ApoCIII is an inhibitor of the activity of LPL (13), which metabolizes TG in VLDL and facilitates its clearance from plasma. In addition, ApoCIII reduces the plasma clearance of VLDL and LDL by inhibiting their interaction with hepatic lipoprotein receptors (14,15).

There is considerable information regarding the effects of apoCIII on lipoprotein metabolism (10,16,17). Statins have been suggested to lower plasma apoCIII levels. In 27 patients with primary hypertriglyceridemia, administration of 20–40 mg atorvastatin for four weeks reduced plasma apoCIII levels by 18–30% (18). However, little information is available regarding the association between microalbuminuria (MAU) and circulating apoCIII levels in DN. To enhance the understanding of this issue, the present study aimed to assess serum apoCIII levels and the association between apoCIII levels and MAU, and to evaluate the effectiveness of statins in lowering apoCIII levels and therefore decreasing MAU in type 2 diabetes.

## Materials and methods

### Patients

Subjects with type 2 diabetes (n=120) aged 48–66 years were recruited from Hebei General Hospital (Shijiazhuang, China). A group of 60 healthy subjects aged 47–65 years (30 male and 30 female) was selected as control subjects. The study was approved by the Institutional Ethic Committee of Hebei General Hospital and written informed consent was obtained from all subjects. Written informed consent was obtained from all subjects. The baseline characteristics of the type 2 diabetes and type 2 DN groups are shown in Table I. The patients with DN were administered atorvastatin (20 mg) daily. Patients were interviewed every four weeks during treatment to assess drug adherence up to a total of 16 weeks.

### Samples

A blood sample was collected following an overnight fast of ≥8 h and prior to insulin administration from the 120 patients with type 2 diabetes and the 60 healthy subjects. Fasting blood samples were taken for the measurement of serum glucose, glycosylated hemoglobin (HbA1c) and the lipid profile. Timed urine samples (24 h) were collected from all patients for the determination of MAU. Fasting blood glucose (FBG) was determined on a Beckman CX9 automatic analyzer (Beckman Coulter, Miami, FL, USA) using a glucose oxidase method. HbA1c levels were determined by high-performance liquid chromatography, using the BioRad Variant Hemoglobin Analyzer (Bio-Rad, Hercules, CA, USA).

For the lipoprotein studies, blood was placed on ice in polypropylene tubes containing a solution of lipoprotein preservatives comprising 2.8 mmol/l EDTA, 62 μmol/l chloramphenicol, 50 μg/ml gentamycin sulfate, 10 mmol/l ɛ-aminocaproic acid and 100 mmol/l 5,5′-dithiobis-(2-nitrobenzoic acid) (final concentrations). Samples were immediately centrifuged at 1,008 × g for 25 min to sediment blood cells and were subsequently stored at −80°C until analysis. Total cholesterol (TC) and TG levels were determined using the enzymatic colorimetric method on the Beckman CX9 automatic analyzer and HDL-cholesterol (HDL-C) was measured using a direct enzymatic HDL-C method based on polyethylene glycol-modified enzymes on the Beckman CX9 automatic analyzer. VLDL-cholesterol (VLDL-C) and LDL-cholesterol (LDL-C) levels were estimated using the Friedewald formula (19). ApoCIII concentration in the serum was determined using a competitive ELISA method (Bioworld Technology, Shanghai, China) (18). The standard curve was fitted to a four-parameter sigmoidal curve. The inter- and intra-assay variability for the assay was <7%.

Body mass index (BMI) and blood pressure (BP) were measured for all the subjects. Obesity was defined as BMI ≥25 kg/m^2^. Hypertension was defined as BP >140/90 mmHg or the administration of anti-hypertensive drug treatment. Fasting blood samples were measured for biochemistry and metabolic profile analysis. Dyslipidemia was defined as TG ≥1.7 mmol/l and/or HDL-C <1.1 mmol/l in males and <1.3 mmol/l in females and/or LDL-C > 2.6 mmol/l or treatment with lipid-lowering drugs. DN was defined as MAU ≥30 mg/24 h in two collections of timed urine samples (24 h) and serum creatinine (Cr) ≤132 μmol/l. Patients with urinary tract infection, hematuria shown by urine microscopy or obstructive uropathy shown by kidney ultrasound suggestive of non-diabetes-related causes were excluded. Type 2 diabetic patients without DN had serum Cr ≤132 μmol/l and MAU <30 mg/24 h in two collections of timed urine samples. Control subjects without diabetes had no known history of diabetes, exhibited normal glucose tolerance at the 75 g oral glucose tolerance test (1998 World Health Organization criteria) (20) and had normal blood biochemistry. The blood and urine samples of patients with type 2 DN were collected after 16 weeks of therapy.

### Statistical analysis

Analyses were performed using SPSS 17.0 for Windows (SPSS, Inc., Chicago, IL, USA). Data are expressed as percentages or as the mean ± standard deviation. The mean differences of continuous variables between the type 2 diabetes and type 2 DN groups were analyzed using the Student’s t-test. The mean differences of categorical variables between these two groups were analyzed using the χ^2^ test. The mean differences among groups were analyzed using analysis of variance, and the Newman-Keuls post hoc test was used in the event of a significant F-ratio. Pearson correlation was performed between MAU and the other variables. Logistic regression analysis was used to assess the association between MAU and other clinically relevant variables, where the clinical variables were considered as independent and MAU as dependent. Estimated odds ratios (ORs) are expressed with their 95% confidence intervals (95% CIs). All significant tests were two-sided and were considered statistically significant at P<0.05.

## Results

### Comparisons of basic clinical data

Compared with the type 2 diabetes group, the type 2 DN group showed a longer course of disease (P<0.01). The stroke and retinopathy rates in the patients with type 2 DN were significantly higher than those in patients without DN (P<0.05). However the occurrence rate of coronary heart disease exhibited no statistical difference between the two groups (P>0.05) (Table I).

### Comparisons of BP, BMI, HbA1c, FBG, lipid profile, apoCIII and MAU

Compared with the control group, systolic BP (SBP), diastolic BP (DBP), BMI and levels of HbA1c, FBG, TG, VLDL-C, apoCIII and MAU were markedly elevated in the type 2 diabetes and type 2 DN groups (P<0.01), while HDL-C levels were decreased significantly (P<0.01). All patients with type 2 DN showed significantly elevated TC and LDL-C levels (P<0.05) (Table II). Although the BMI and levels of HbA1c, FBG, TG, TC, LDL-C and VLDL-C were elevated in the type 2 DN group compared with those in the type 2 diabetes group, the differences were not significant (P>0.05). However, the levels of SBP, DBP, apoCIII and MAU in this group were more notably increased (P<0.01) (Table II).

### Associations between MAU and age, gender, course of the disease, BP, BMI, HbA1c, FBG, lipid profile and apoCIII in type 2 diabetes and type 2DN patients

MAU was significantly positively correlated with the course of the disease, SBP, DBP, BMI, HbA1c, FBG, TG, TC, LDL-C, VLDL-C and apoCIII, whereas negatively correlated with HDL-C. MAU exhibited no association with age or gender (Table III).

### Multiple regression analysis for MAU in type 2 diabetes and type 2DN patients

To further clarify the independent associations of age, gender, course of the disease, BP, BMI, HbA1c, FBG, lipid profile and apoCIII with MAU, logistic regression analysis was performed. This revealed that only apoCIII was independently associated with MAU (OR, 1.100; 95% CI, 1.037–1.153; P<0.001). The other variables were not independent predisposing factors for MAU in type 2 diabetes (Table IV).

### Atorvastatin improves the lipid profile and MAU levels in patients with type 2 DN

After 16 weeks of atorvastatin administration in patients with type 2 DN, levels of TG, TC, LDL-C, VLDL-C and apoCIII were significantly decreased compared with those prior to treatment, while HDL-C levels were found to have increased markedly (P<0.05). MAU levels were observed to have decreased significantly (P<0.01) (Table V).

## Discussion

There is currently a global epidemic of type 2 diabetes mellitus (DM), which accounts for 40–50% of all new cases of ESRD. Among the clinical signs of nephropathy, the appearance of low but abnormal levels (>30 mg/day) of albumin in the urine, known as MAU, is first to occur. MAU is a leading cause of DM-related morbidity and mortality (21). In type 2 diabetes, lipotoxicity and glucotoxicity increase the risk of diabetic micro- and macrovascular complications (3,22).

In the majority of type 2 diabetic patients, insulin resistance is a key pathophysiological feature. Lipases are insulin-sensitive enzymes that hydrolyze TG in TRL particles. Diabetic dyslipidemia is typically characterized by high TG and low HDL-C levels. The high TG levels in turn can alter the composition of LDL-C and HDL-C, making these lipid particles more atherogenic (23). Nephritis can be exacerbated through increased TG levels and oxidized remnant lipoprotein particles, which induce mesangial cells to proliferate and secrete cytokines (24). Following the initiation of proteinuria, the loss of lipoprotein particles in the urine can enhance their synthesis in the liver. Furthermore, the lipoprotein particles can cause renal damage by binding to glomerular basement membranes and renal tubular cells. This can initiate a vicious cycle of proteinuria and dyslipidemia (22). Patients with renal disease, whether associated with diabetes or not, have increased TG and remnant lipoprotein levels and decreased LPL and HL activity (3). In addition to the effects of lipotoxicity on the kidney, increased von Willebrand factor and decreased heparin-releasable LPL levels lead to vascular endothelial damage. Reduced lipase binding to the damaged endothelium may further increase TG levels in DN (3).

The present study showed that BP, BMI and levels of HbA1c, FBG, TG, VLDL-C, apoCIII and MAU were markedly elevated in the type 2 diabetes and type 2 DN groups compared with those in the control group (P<0.01), while HDL-C levels were decreased significantly (P<0.01). These findings were consistent with those in other studies (3,23). All patients with type 2 DN showed significantly elevated BP, apoCIII, MAU, course of the disease and stroke and retinopathy rates compared with the patients with type 2 diabetes (P<0.01).

ApoCIII is an effective inhibitor of LPL activity and is present in TRLs and HDLs (13). Sections of apoCIII can be exchanged between these lipoproteins (24). Upon an elevation in TRL concentration, ApoCIII is transferred from HDLs to TRLs, which causes a decrease in HDL apoCIII concentration. Following the degradation of TG in TRLs by LPLs, apoCIII transfers back to the HDLs, such that clearance of the TRL particles may proceed uninhibited (25). In a study by Klein *et al* (26), an independent positive association between apoCIII levels and type 1 diabetes-related microvascular complications was found in the cohort of patients.

The present study revealed that MAU was significantly positively correlated with the course of the disease, SBP, DBP, BMI and HbA1c, FBG, TG, TC, LDL-C VLDL-C and apoCIII levels, whereas negatively correlated with HDL-C levels (P<0.05). MAU exhibited no association with age and gender (P>0.05). Logistic regression analysis revealed that only apoCIII was independently associated with MAU (OR, 1.100; 95% CI, 1.037–1.153; P<0.001). Other factors were not independent predisposing factors for MAU in type 2 diabetes.

Atorvastatin is a potent 3-hydroxy-3-methylglutaryl-coenzyme A reductase inhibitor that has been shown to effectively reduce total plasma TC and TG levels. Statins have become the preferred agents as lipid-lowering drugs in diabetic patients (27). Statins have been reported to exert cardioprotective and antioxidant effects, regardless of their effect on LDL-C reduction (28). In a study of 305 patients with primary hypercholesterolemia, atorvastatin and pravastatin reduced levels of lipoprotein particles containing apoCIII and apoB by 30% (29). The present study indicated that, by the end of 16 weeks, atorvastatin decreased TG, TC, LDL-C, VLDL-C and apoCIII levels significantly (P<0.05), while increasing HDL-C markedly (P<0.05) in patients with type 2 DN. A significant decrease in MAU was also observed (P<0.01). Rutter *et al* (30) compared the renal effects of treatment with low- versus high-dose atorvastatin in patients with type 2 DM and optimally managed early renal disease. No statistical difference in renal function was identified between the high- and low-dose atorvastatin treatment groups over two years. The beneficial effect of a glomerular filtration rate of <1.6 ml/min/1.73 m^2^/year, as estimated using the Modification of Diet in Renal Disease equation, was observed. However, such an effect may have been due to blood pressure management and/or a renin-angiotensin system blocker use (30,31). With regard to the present findings, the decrease in MAU in patients with type 2 DN may have been due to comprehensive treatment. In conclusion, in type 2 diabetes, circulating apoCIII levels were independently correlated with MAU. Atorvastatin improved the lipid profile in patients and may contribute to decreasing MAU levels in type 2 DN.

## Figures and Tables

**Table I:** Baseline characteristics of the two groups.

Variable	Type 2 diabetes	Type 2 DN	P-value
Number (male/female)	60 (28/32)	60 (33/27)	0.361
Age, years^a^	55.3±9.8	56.0±9.5	0.692
Course of disease, years^a^	5.8±3.1	7.8±3.0	0.001
Coronary heart disease, %	8.3	13.3	0.378
Stroke, %	5.0	16.7	0.040
Retinopathy, %	28.3	55.0	0.003

a Values are presented as the mean ± standard deviation.

DN, diabetic nephropathy.

**Table II:** Comparisons of BP, BMI, HbA1c, FBG, lipid profile, apoCIII and MAU.

Variable	Control	Type 2 diabetes	Type 2 diabetic nephropathy
SBP, mmHg	121.55±10.61	131.53±10.72^a^	152.1±11.80^a^,^b^
DBP, mmHg	72.12±8.52	74.48±7.00	83.10±9.34^a^,^b^
BMI, kg/m^2^	20.12±2.07	24.13±2.02^a^	24.79±1.83^a^
HbA1c, %	4.91±1.08	7.37±1.11^a^	7.78±1.22^a^
FBG, mmol/l	4.41±0.38	8.23±2.29^a^	8.91±2.51^a^
TG, mmol/l	1.26±0.51	1.57±0.56^a^	1.75±0.44^a^
TC, mmol/l	4.35±0.91	4.73±1.15	5.12±1.26^a^
HDL-C, mmol/l	1.13±0.29	0.97±0.28^a^	0.88±0.23^a^
LDL-C, mmol/l	2.66±0.72	2.95±0.84	3.15±0.86^a^
VLDL-C, mmol/l	0.51±0.24	0.72±0.31^a^	0.83±0.37^a^
apoCIII, μg/ml	10.65±4.58	14.67±5.07^a^	19.48±4.86^a^,^b^
MAU, mg/24 h	6.61±1.15	12.11±5.19	229.76±41.06^a^,^b^

Values are presented as the mean ± standard deviation (n=60/group).

a P<0.01 vs. control;

b P<0.01 vs. type 2 diabetes.

SBP, systolic blood pressure; DBP, diastolic blood pressure; BMI, body mass index; HbA1c, glycosylated hemoglobin; FBG, fasting blood glucose; TG, triglyceride; TC, total cholesterol; HDL-C, high-density lipoprotein-cholesterol; LDL-C, low-density lipoprotein-cholesterol; VLDL-C, very low-density lipoprotein-cholesterol; apoCIII, apolipoprotein CIII; MAU, microalbuminuria.

**Table III:** Associations between microalbuminuria and age, gender, course of disease, BP, BMI, HbA1c, FBG, lipid profile and apoCIII in type 2 diabetes and type 2 DN patients.

Variable	*r*	P-value
Age, years	0.023	0.400
Gender	0.031	0.359
Course of disease, years	0.267	0.002
SBP, mmHg	0.429	<0.001
DBP, mmHg	0.438	<0.001
BMI, kg/m^2^	0.204	0.013
HbA1c, %	0.179	0.025
FBG, mmol/l	0.164	0.037
TG, mmol/l	0.208	0.011
TC, mmol/l	0.156	0.044
HDL-C, mmol/l	−0.194	0.020
LDL-C, mmol/l	0.193	0.021
VLDL-C, mmol/l	0.168	0.036
apoCIII, μg/ml	0.501	<0.001

SBP, systolic blood pressure; DBP, diastolic blood pressure; BMI, body mass index; HbA1c, glycosylated hemoglobin; FBG, fasting blood glucose; TG, triglyceride; TC, total cholesterol; HDL-C, high-density lipoprotein-cholesterol; LDL-C, low-density lipoprotein-cholesterol; VLDL-C, very low-density lipoprotein-cholesterol; apoCIII, apolipoprotein CIII.

**Table IV:** Multiple regression analysis for microalbuminuria in type 2 diabetes and type 2 DN patients..

Variable	OR	95% CI	P-value
Age, years	1.015	0.985–1.045	0.323
Gender	0.990	0.544–1.803	0.974
Course of disease, years	1.045	0.948–1.153	0.374
SBP, mmHg	1.031	0.996–1.068	0.082
DBP, mmHg	0.984	0.938–1.032	0.498
BMI, kg/m^2^	1.086	0.919–1.283	0.355
HbA1c, %	1.048	0.798–1.375	0.738
FBG, mmol/l	0.991	0.866–1.135	0.900
TG, mmol/l	0.934	0.489–1.781	0.835
TC, mmol/l	1.106	0.870–1.406	0.412
HDL-C, mmol/l	1.645	0.617–4.386	0.320
LDL-C, mmol/l	1.117	0.133–1.704	0.607
VLDL-C, mmol/l	1.237	0.497–3.080	0.648
apoCIII, μg/ml	1.100	1.037–1.153	<0.001

SBP, systolic blood pressure; DBP, diastolic blood pressure; BMI, body mass index; HbA1c, glycosylated hemoglobin; FBG, fasting blood glucose; TG, triglyceride; TC, total cholesterol; HDL-C, high-density lipoprotein-cholesterol; LDL-C, low-density lipoprotein-cholesterol; VLDL-C, very low-density lipoprotein-cholesterol; apoCIII, apolipoprotein CIII; OR, odds ratio; CI, confidence interval.

**Table V:** Mean differences of changes in the lipid profile and MAU after 16 weeks of atorvastatin administration in patients with type 2 DN.

Variable	Before administration	After administration	*t*	P-value
TG, mmol/l	1.725±0.44	1.45±0.238	3.7306	0.0002
TC, mmol/l	5.12±1.26	4.34±1.03	3.7125	0.0002
HDL-C, mmol/l	0.88±0.23	1.08±0.20	5.0827	<0.0001
LDL-C, mmol/l	3.15±0.86	2.64±0.75	3.4620	0.0005
VLDL-C, mmol/l	0.83±0.37	0.62±0.31	3.3699	0.0008
apoCIII, μg/ml	19.48±4.86	12.75±4.01	8.2736	<0.0001
MAU, mg/24 h	229.76±41.06	110.48±27.86	18.6204	<0.0001

Values are presented as the mean ± standard deviation (n=60). TG, triglyceride; TC, total cholesterol; HDL-C, high-density lipoprotein-cholesterol; LDL-C, low-density lipoprotein-cholesterol; VLDL-C, very low-density lipoprotein-cholesterol; apoCIII, apolipoprotein C-III; MAU, microalbuminuria.

## References

[1] Molitch ME, DeFronzo RA, Franz MJ, Keane WF, Mogensen CE, Parving HH, Steffes MW, American Diabetes Association (2004). Nephropathy in diabetes. Diabetes Care.

[2] Ravid M, Brosh D, Ravid-Safran D, Levy Z, Rachmani R (1998). Main risk factors for nephropathy in type 2 diabetes mellitus are plasma cholesterol levels, mean blood pressure, and hyperglycemia. Arch Intern Med.

[3] Hirano T (1999). Lipoprotein abnormalities in diabetic nephropathy. Kidney Int Suppl.

[4] Boyle KE, Phillips MC, Lund-Katz S (1999). Kinetics and mechanism of exchange of apolipoprotein C-III molecules from very low density lipoprotein particles. Biochim Biophys Acta.

[5] Breyer ED, Le NA, Li X, Martinson D, Brown WV (1999). Apolipoprotein C-III displacement of apolipoprotein E from VLDL: effect of particle size. J Lipid Res.

[6] Jong MC, Hofker MH, Havekes LM (1999). Role of ApoCs in lipoprotein metabolism: functional differences between ApoC1, ApoC2, and ApoC3. Arterioscler Thromb Vasc Biol.

[7] Campos H, Perlov D, Khoo C, Sacks FM (2001). Distinct patterns of lipoproteins with apoB defined by presence of apoE or apoC-III in hypercholesterolemia and hypertriglyceridemia. J Lipid Res.

[8] Davidsson P, Hulthe J, Fagerberg B, Olsson BM, Hallberg C, Dahllöf B, Camejo G (2005). A proteomic study of the apolipoproteins in LDL subclasses in patients with the metabolic syndrome and type 2 diabetes. J Lipid Res.

[9] Schonfeld G, George PK, Miller J, Reilly P, Witztum J (1979). Apolipoprotein C-II and C-III levels in hyperlipoproteinemia. Metabolism.

[10] Talmud PJ, Humphries SE (1997). Apolipoprotein C-III gene variation and dyslipidaemia. Curr Opin Lipidol.

[11] Sacks FM, Alaupovic P, Moye LA, Cole TG, Sussex B, Stampfer MJ, Pfeffer MA, Braunwald E (2000). VLDL, apolipoproteins B, CIII, and E, and risk of recurrent coronary events in the Cholesterol and Recurrent Events (CARE) trial. Circulation.

[12] Lee SJ, Campos H, Moye LA, Sacks FM (2003). LDL containing apolipoprotein CIII is an independent risk factor for coronary events in diabetic patients. Arterioscler Thromb Vasc Biol.

[13] Ginsberg HN, Le NA, Goldberg IJ, Gibson JC, Rubinstein A, Wang-Iverson P, Norum R, Brown WV (1986). Apolipoprotein B metabolism in subjects with deficiency of apolipoproteins CIII and AI. Evidence that apolipoprotein CIII inhibits catabolism of triglyceride-rich lipoproteins by lipoprotein lipase in vivo. J Clin Invest.

[14] Ebara T, Ramakrishnan R, Steiner G, Shachter NS (1997). Chylomicronemia due to apolipoprotein CIII overexpression in apolipoprotein E-null mice. Apolipoprotein CIII-induced hypertriglyceridemia is not mediated by effects on apolipoprotein E. J Clin Invest.

[15] Sehayek E, Eisenberg S (1991). Mechanisms of inhibition by apolipoprotein C of apolipoprotein E-dependent cellular metabolism of human triglyceride-rich lipoproteins through the low density lipoprotein receptor pathway. J Biol Chem.

[16] Jong MC, Hofker MH, Havekes LM (1999). Role of ApoCs in lipoprotein metabolism: functional differences between ApoC1, ApoC2, and ApoC3. Arterioscler Thromb Vasc Biol.

[17] Shachter NS (2001). Apolipoproteins C-I and C-III as important modulators of lipoprotein metabolism. Curr Opin Lipidol.

[18] Le NA, Innis-Whitehouse W, Li X, Bakker-Arkema R, Black D, Brown WV (2000). Lipid and apolipoprotein levels and distribution in patients with hypertriglyceridemia: effect of triglyceride reductions with atorvastatin. Metabolism.

[19] Friedewald WT, Levy RI, Fredrickson DS (1972). Estimation of the concentration of low-density lipoprotein cholesterol in plasma, without use of the preparative ultracentrifuge. Clin Chem.

[20] Alberti KG, Zimmet PZ (1998). Definition, diagnosis and classification of diabetes mellitus and its complications. Part 1: diagnosis and classification of diabetes mellitus provisional report of a WHO consultation. Diabet Med.

[21] Genuth SM (1995). The case for blood glucose control. Adv Intern Med.

[22] Cooper ME (1998). Pathogenesis, prevention, and treatment of diabetic nephropathy. Lancet.

[23] Brunzell JD, Ayyobi AF (2003). Dyslipidemia in the metabolic syndrome and type 2 diabetes mellitus. Am J Med.

[24] Batal R, Tremblay M, Barrett PH, Jacques H, Fredenrich A, Mamer O, Davignon J, Cohn JS (2000). Plasma kinetics of apoC-III and apoE in normolipidemic and hypertriglyceridemic subjects. J Lipid Res.

[25] Barr SI, Kottke BA, Mao SJ (1981). Postprandial exchange of apolipoprotein C-III between plasma lipoproteins. Am J Clin Nutr.

[26] Klein RL, McHenry MB, Lok KH, Hunter SJ, Le NA, Jenkins AJ, Zheng D, Semler A, Page G, Brown WV, Lyons TJ, Garvey WT, DCCT/EDIC Research Group (2005). Apolipoprotein C-III protein concentrations and gene polymorphisms in Type 1 diabetes: associations with microvascular disease complications in the DCCT/EDIC cohort. J Diabetes Complications.

[27] Davidson M (2008). A review of the current status of the management of mixed dyslipidemia associated with diabetes mellitus and metabolic syndrome. Am J Cardiol.

[28] Takemoto M, Liao JK (2001). Pleiotropic effects of 3-hydroxy-3-methylglutaryl coenzyme a reductase inhibitors. Arterioscler Thromb Vasc Biol.

[29] Dallongeville J, Fruchart JC, Maigret P, Bertolini S, Bon GB, Campbell MM, Farnier M, Langan J, Mahla G, Pauciullo P, Sirtori C (1998). Double-blind comparison of apolipoprotein and lipoprotein particle lowering effects of atorvastatin and pravastatin monotherapy in patients with primary hypercholesterolemia. J Cardiovasc Pharmacol Ther.

[30] Rutter MK, Prais HR, Charlton-Menys V, Gittins M, Roberts C, Davies RR, Moorhouse A, Jinadev P, France M, Wiles PG, Gibson JM, Dean J, Kalra PA, Cruickshank JK, Durrington PN (2011). Protection Against Nephropathy in Diabetes with Atorvastatin (PANDA): a randomized double-blind placebo-controlled trial of high- vs. low-dose atorvastatin. Diabet Med.

[31] Levey AS, Coresh J, Greene T, Marsh J, Stevens LA, Kusek JW, Van Lente F, Chronic Kidney Disease Epidemiology Collaboration (2007). Expressing the modification of diet in renal disease study equation for estimating glomerular filtration rate with standardized serum creatinine values. Clin Chem.

